# Botanical Description of British Plants

**Published:** 1808-04

**Authors:** 


### *Botanical Description of British Plants.*

[ Continued from our last, pp. 257—262. ]

Syngenesia. Superflua.

17. TANACETUM. *T. vulgare.*

*Ang.* Tansy.

*Gen. Desc.* Recept. naked. Down 0. Cal. hemispherical, rical, tiled. Florets of the circumference 3-cleft, narrow, strap-shaped, sometimes wanting.

*Spec. Desc.* *Leaves* doubly winged, cut, acutely serrated. *Stem* reddish. *Flowers* in a corymbus, yellow. *Herm.* 5-cleft, *fem.* 3-cleft. *Meadows, river banks, swamps.* Bl. June.

*Use* The leaves and flowers have a strong smell, and a bitter, somewhat aromatic taste; the flowers are stronger than the leaves; the tincture made from the latter is of a fine green; from the flowers, of a bright pale yellow colour. —*Lewis.* The virtues of tansy are said, by Bergius, to be tonic, stomachic, anthelmintic, emmenagogue, and resolvent, qualities usually attributed to bitters of the warm or aromatic kind. It has been much used as a vermifuge, and testimonies of its efficacy are given by many respectable physicians. The seeds as well as the leaves have been employed with this intention, and substituted for those of santonicum. It is said by Dr. Clark, that this plant has been found, in Scotland, of great service in various cases of gout; *Ess. and Obs. Phys. and Lit. iii. p.* 435; and Dr. Cullen reports, that he has known several who have taken it without any advantage, and some others who reported, that they had been relieved from the frequency of their gout. *M. M. ii.* 89. Tansy is also recommended in hysteria, especially when this disease is supposed to proceed from menstrual obstructions. It may be given in powder to the quantity of a drachm, or more, for a dose; but it has commonly been taken in infusion, or drank as tea.—*Woodville.* This plant is esteemed good to warm and strengthen the stomach, but it is rarely used as a medicine, though extolled as a good emmenagogue; a drachm of the dried flowers has been found useful in hysterics, arising from suppressions. The seeds and leaves were formerly thought to destroy worms in children, and are useful in cholics and flatulencies. In some parts of Sweden and Lapland, a bath, with a decoction of this plant, is used to assist parturition.—*Lightfoot.* It is a warm deobstruent bitter, and its flavour not ungrateful. The seeds are an excellent vermifuge. The tender leaves are sometimes used to give a colour and flavour to puddings. The Finlanders obtain a green dye from it. If a dead animal substance be rubbed with this plant, the flesh fly will not attack it. Cows and sheep eat it; horses, goats, and swine, refuse it.—*Withering.*

18. Ar-

18. Artemisia. *A. maritima.*

*Ang.* Sea southernwood. Sea wormwood.

*Gen. Desc.* Recept. either slightly hairy or naked. Down, none. Cal. tiled; the scales roundish, closing. Florets radiate, none.

*Spec. Desc.* Whole plant white with thick cotton. *Leaves* many-cleft, varying much in their division; *upper* generally simple, strap-shaped, blunt. *Bunches* on crooked fruit stalks. *Female florets* 3. *Seeds* seldom ripen in gardens. *Sea shores.* Bl. August.

*Use.* Its taste and smell are less unpleasant, its essential oil, which contains the whole of its flavour concentrated, is less ungrateful, and the watery extract less bitter, than those of the common wormwood; hence it is preferred by the London College in those cases where the A. absinthium is supposed to be too offensive to the stomach. But as the efficacy of these plants depends upon their sensible qualities, this species, though its virtues approach to those of the common wormwood, yet from being less powerfully bitter, must be considered in a proportionate degree a less powerful medicine. A conserve of the tops is directed by the *Lond. Pharm.—Woodville.* In its wild state, it smells like marum or camphor, but in our gardens it is less grateful, though still much more so than the common wormwood. It is used as an ingredient in distilled waters, and beat with thrice its weight, it is formed into a conserve. Horses eat it; cows, sheep, and goats refuse it.—*Withering.*

19. Artemisia. *A. absinthium.—A. vulgare.—A. ponticum; Romanum.*

*Ang.* Wormwood. Wormwood-southernwood.

*Gen. Desc.* *As above.*

*Spec. Desc.* *Stem* herbaceous, upright, scored, whitish with very short down. *Leaves* compound, many-cleft, silky, very soft, green above, white shining underneath; the *upper* 3 cleft, or simple, sitting, bluntly spear-shaped; *lower* on long flat leaf-st. *Flowers* somewhat globular, pendent, brownish white. *Recept.* woolly; down as long as the florets. *Spikes* upright.—*Rocky places, rubbish.* Bl. August.

*Use.* The leaves have a strong disagreeable smell; their taste is nauseous, and so intensely bitter as to be proverbial: the flowers are more aromatic and less bitter; and the roots discover an aromatic warmth without any bitterness. This may be considered the principal of the herbaceous bitters; and its virtues are stated by Bergius to be

 antiseptic, antiseptic, antiacid, anthelmintic, resolvent, tonic, and stomachic. It is now chiefly employed with a view to the two last mentioned qualities; but we are told of its good effects in a great variety of diseases, as intermittent fevers, hypochondriasis, obstructions of the liver and spleen, gout, calculi, scurvy, dropsy, worms, &c. It is asserted by Lindestolphe, that by the continued use of this plant, great injury is done to the nervous system, from its narcotic and debilitating effects, which he has experienced upon himself; observing also that he never could taste the extract or essence of wormwood without being immediately affected with head-ache and inflammation of the eyes.

These narcotic effects have, however, been attributed to a peculiar idiosyncrasy, as numerous instances have occurred in which this plant produced a contrary effect, though taken daily for the space of six months. Dr. Cullen thinks that the odour of wormwood is temulentans, that is, giving some confusion to the head; and says, that formerly when it was the fashion to drink purl, that is, ale in which wormwood is infused, it was commonly alleged to be more intoxicating than other ales: this effect has been improperly attributed to its volatile parts, but Dr. C. places it to the account of its narcotic power; and he believes, from several considerations, particularly from the history of the Portland powder, that there is in every bitter, when largely employed, a power of destroying the sensibility and irritability of the nervous power. *M. M. v. ii. p.* 81. Externally, it is used in discutient and antiseptic fomentations. It may be taken in powder, but it is more commonly preferred in infusion: a tincture of the flowers is directed by the Edinb. Pharm. as a light and agreeable bitter, and at the same time, a strong impregnation of the wormwood.—*Woodville.* The leaves and flowers are very bitter; the roots are warm and aromatic. In distillation, a considerable quantity of oil arises, which is used both internally and externally to destroy worms. The leaves resist putrefaction, and are, therefore, a principal ingredient in antiseptic fomentations. An infusion of them is a good stomachic; and, with the addition of fixed alkaline salt, a powerful diuretic in some dropsical cases. Linnæus mentions two cases, wherein an essence prepared from this plant, and taken for a considerable time, prevented the formation of stones in the kidneys or bladder; the patient forbearing the use of wine or acids. (*Amœn. Acad. v. ii. p.* 160.) It might be suspected that, like other bitters, its long continued use must weaken the action action of the nervous system; but in these instances it had no such effect.—The leaves put into sour beer, soon destroy the acescency.—The ashes afford a more pure alkaline salt than most other vegetables, excepting bean stalks, broom, and the larger trees.—The plant steeped in boiling water, and repeatedly applied to a bruise, will remove the pain in a short time, and prevent the swelling and discolouration of the part.—*Withering.* Its bitterness is so great, that it communicates a bitter taste to the flesh and milk of cows and sheep that feed on it.—*Linn. Flor. Succ.* And the milk of a woman, who took the extract, became extremely bitter. *Act. Hafn. ii. p.* 165. An infusion of it will make a woman's milk bitter.—*With.* Turkeys are fond of it; horses and goats are not fond of it; cows and swine refuse it.—*Ib.*

20. Artemisia. *A. vulgaris.*
*Ang.* Mugwort. Southernwood.
*Gen. Desc.* *As above.*

*Spec. Desc.* *Leaves* wing-cleft, flat, cottony underneath, cut; wings oval-spear-sh. serrated, almost lobed, *termin.* large, three-lobed. *Bunches* simple, bending, axillary. *Flor. of circumf.* 5, longer than the cal. *Bloss.* purplish. *Recept.* naked. *Cal.* woolly. *Stems* ascending, angular, scored, reddish.—*Borders of fields, rubbish.* Bl. August.

*Use.* The leaves have a light agreeable smell. This plant is little used now for medicinal purposes, but it was highly esteemed by the ancients, especially as an emmenagogue, both taken internally and employed in the way of fomentation: the Chinese women are said to use a poultice of the leaves, mixed with rice and sugar, which in cases of amenorrhœa and hysteria, instar bellarii ingerunt. Its powers, however, as an antihysteric, or uterine, are very weak, and it has been expunged from the London Mat. Med.—From the dryed tops and leaves of this plant, a substance is prepared in Japan, called Moxa, by beating and rubbing them between the hands till only the fine internal lanuginous fibres remain, which are then combed and formed into little cones. These, used as cauteries, are greatly celebrated in Eastern countries for preventing and curing many disorders: but chronic rheumatisms, gouty, and some other painful affections of the joints, seem to be the chief complaints for which they can be rationally employed. It is thus applied: the part affected being previously moistened, a cone of the moxa is laid, which being set on fire at the apex, gradually burns down to the skin, where it produces a dark coloured spot; by repeating the

 process process several times, an eschar is formed of any desired extent, and this on separation leaves an ulcer, which is kept open or healed, as circumstances may require. *(See Kæmpf. Am. Exot. p.* 502.*)* It is related by Abbé Grosier, *(Hist. of China,)* that mirrors of ice or metal were used for the purpose of igniting the moxa. The practice of cauterizing the part affected is very ancient; Hippocrates used not only iron but flax, and also a species of fungus: the Laplanders prepare and use the Agaric in a similar way, as the Japanese do their moxa. The Egyptians produced the same effect by means of cotton or linen cloth, *(Prosp. Alpin.)* and in Spain a moxa is prepared from a species of Echinops. This is not, however, the species of A. from which the eastern moxa is prepared, but that made from this plant in Germany was found to answer very well. *Woodville.* The Japanese moxa is no other than a spongy inflammable substance prepared from the medulla of the stalks of this plant.—An infusion of the plant in white wine, or a bath made of it, has been always esteemed an emmenagogue, and useful in difficult parturition. In Sweden, a decoction of the leaves is used by the country people for the ague.—*Lightfoot.* And in England also. To a woman who had been affected with hysteric fits for many years, Dr. Home gave 1 dr. of the leaves powdered four times a day; and in a few days the fits ceased. Assafœtida and æther had been tried to no purpose—*Withering.* It is used by the Chinese in healing wounds, by an application of the fresh plant bruised. *Osbeck. i.* 394. And the ancient Chinese are said to have made paper and a kind of cloth from the down of the leaves.—*Grosier.* In some countries it is used as a culinary aromatic.—*With.* And in Scotland the highlanders employ the leaves, when young and tender, as a pot-herb.—*Lightf.* Neither horses, cows, nor goats are fond of it; sheep and swine refuse it.—*Linn.* But Dr. Anderson says that sheep are very fond of it, devouring it with great greediness, especially the roots, which seem to form a most delicate morsel.—*Withering.*

21. GNAPHALIUM. *G. germanicum.—Filago germanica.*

*Ang.* Common cudweed. Chafeweed.

*Gen. Desc.* Recept. naked. Down hair-like, or feathered. Cal. tiled. Scales at the edge roundish, skinny, coloured.

*Spec. Desc. Panicle* forked. *Flowers,* roundish, axillary, hairy, sitting, yellowish brown; *fem. flor.* not within the common cal. but between its scales. *Leaves* acute, strap-spear-shaped, sitting, waved, cottony. *Stems* several, ral, the thickest in the centre, upright, branched. *Branches* horizontal, rising above the stems. *Cal.* five-cornered, *outer scales* woolly, *inner* skinny, spear-shaped, pointed. *Stam.* 4. *Germ.* rough, downy. *Seeds* oblong.—*Barren pastures, roads.* Bl. July, August.

*Use.* This plant is given to cattle for the *bloody flux*; and it has been tried with success in similar complaints of the human body. A horse eat it.—*Withering.*

22. Tussilago. *T farfara. T. vulgaris. Bechium. Petasites.*

*Ang.* Colt's foot.

*Gen. Desc.* Recept. naked. Down hair-like. Calyx scales equal, as tall as the surface of the florets, somewhat membranaceous.

*Spec. Desc.* *Stalk* with 1 flower, tiled, numerous, solitary or in clusters, 3 to 5 inches high, lengthening after flowering, cottony; clothed with scales spear-shaped, embracing, green mixed with brown. *Leaves* somewhat heart-shaped, angular, toothed, appearing as the flowers go off, green above with reddish veins, white and cottony underneath. *Leaf-st.* long, reddish brown. *Root* creeping. *Flowers* yellow, while in blossom upright, then pendant; when the down of the seeds expands, upright again. *Flor. of the circumf.* v. narrow, in 2 or 3 rows, as long as the calyx, expanding. *Fl. of the centre* tubular, swelling upwards, five-cleft, spear-shaped, bent back.—*Moist stiff soil, clay, limestone rubbish.* Bl. March, April.

*Use.* The sensible qualities are very inconsiderable: it has a rough mucilaginous taste. The leaves have always been of great fame as possessing demulcent and pectoral virtues; it is therefore esteemed useful in pulmonary consumption, coughs, asthmas, and in various catarrhal symptoms. Dr. Percival found it useful also in hectic diarrhœas. *(Ess. Med. &c. v. ii. p. 224.)* The juice liberally drank has been found beneficial in calculous complaints. *(Comm. Lit. Nor. 1736.)* Fuller recommends it as a valuable medicine in scrophula. *(Med. Gymn. p. 84;)* and Dr. Cullen, who allows it no powers as an expectorant or demulcent, found it useful in some strumous affections. It may be used as tea, or given in the way of infusion, to which liquorice root or honey may be a useful addition. Every part of the plant has been medicinally employed, but more usually the leaves, which are the principal ingredient or basis of the British herb tobacco.—*Woodville.* The leaves smoked in the manner of tobacco, or a syrup or decoction of them and the flowers is recommended in

 coughs coughs and other disorders of the breast and lungs. Practice seems however almost to have rejected it.—*Lightfoot.* It was perhaps not without reason that the leaves were formerly much used in coughs and consumptive complaints; for Dr. Cullen has found them of considerable service in scrophulous cases. *(See M. M. p.* 456.*)* A girl is mentioned by Fuller, (l. c.) who, with twelve scrophulous sores, was cured by drinking daily for above four months, as much as she could of a decoction of the leaves made so strong as to be sweetish and glutinous.—The downy substance on the under surface of the leaves wrapped in a rag, dipped in a solution of saltpetre, and dried in the sun, (or boiled in a lixivium with a little saltpetre, *Lightfoot,*) makes the best tinder. This is the first plant that vegetates in marle or limestone rubble: it may be destroyed by cutting off the crown of the root in March.—Sheep and goats eat it; cows are not fond of it; horses and swine refuse it.—*Withering.*

23. Senecio. *S. vulgaris.*

*Ang.* Groundsel. Simson.

*Gen. Desc.* Recept. naked. Down hair-like, long. Cal. conical, double; scales as if dead at the ends.

*Spec. Desc.* *Florets* all tubular, all hermaphrodite. *Leaves* winged-indented, embracing. *Flowers* scattered, yellow, terminating.—*Cultivated ground, rubbish, court yards.* Bl. April, Sept.

*Use.* In the highlands of Scotland this plant is used externally in cataplasms as a cooler, and to bring on suppurations. A strong infusion of it also acts as an emetic.—*Lightfoot.* The bruised leaves are a good application to boils.—*Withering.* As an emetic, this plant is said to be particularly useful in agues.—*H. B.* The seeds are very acceptable to linnets and goldfinches when confined in a cage.—Goats and swine eat it; cows are not fond of it; horses and sheep refuse it.

24. Senecio. *S. jacobæa.*

*Ang.* Rag wort. Groundsel. Seggram. St. James's wort.

*Gen. Desc.* As above.

*Spec. Desc.* *Leaves* lyre-shaped, almost winged; segm. variously lobed and toothed, sometimes again wing-cleft. *Stem* upright, often cottony; cylind. scored, tinged with purple. *Bloss.* deep yellow. *Flor. of circumf.* 13, expanding, *of cent.* 60. *Cal. scales* 13 or 15. *Var.* 2. *Fl. without rays.* *V.* 3. *plant hoary.*—*Meadows, pastures, road-sides.* Bl. July.

Use.

*Use.* This is said to be useful for old swellings.—*H. B.* If it be gathered before the flowers open, and used fresh, it dyes wool of a full green; but the colour is apt to fade. If woollen cloth be boiled in alum-water, and then in a decoction of the flowers, it takes a beautiful deep yellow.—Horses and sheep refuse it; cows are not fond of it, but eat it when young.—*Withering.*

25. SOLIDAGO. *S. virga-aurea.*

*Ang.* Goldenrod. Woundwort.

*Gen. Des.* Recept. naked. Down hair-like. Florets of the circumference about 5. Calyx scales tiled, laid close.

*Spec. Desc.* *Stem.* serpentine, branched, 1 to 4 feet high. *Leaves* mostly sitting. *Root-l.* serrated. *Stem-l.* generally entire, rough, slightly hairy, dark green underneath, above sea-green, with a net work of veins; *upper* alternate. *Flowers* in crowded panicles of 6 to 8 fl. yellow; *flor. of circumf.* 6 to 8, bent back, with 4 or 5 faint longitudinal scores; *flor. of cent.* segments bent back. *Cal.* scales unequal, spear-shaped, a green line along the back, whitish shining membranaceous edges. *Seed* brown, on one side convex, on the other flat.—*Woods, heaths, hedges, copses.* BL. August, Sept.

*Use.* The leaves are astringent and bitter; and are esteemed as a good vulnerary and a diuretic: they are recommended in the stone and gravel, and in ulcers of the kidneys and bladder, 3 drachms of the powder being taken every eighth hour.—*Lightfoot.*

( To be continued. )

